# Omega-3 PUFAs Lower the Propensity for Arachidonic Acid Cascade Overreactions

**DOI:** 10.1155/2015/285135

**Published:** 2015-08-02

**Authors:** Bill Lands

**Affiliations:** 6100 Westchester Park Drive, Apartment 1219, College Park, MD 20740, USA

## Abstract

A productive view of the benefits from omega-3 (n-3) nutrients is that the dietary essential omega-6 (n-6) linoleic acid has a very narrow therapeutic window which is widened by n-3 nutrients. The benefit from moderate physiological actions of the arachidonic acid cascade can easily shift to harm from excessive pathophysiological actions. Recognizing the factors that predispose the cascade to an unwanted overactivity gives a rational approach for arranging beneficial interactions between the n-3 and n-6 essential nutrients that are initial components of the cascade. Much detailed evidence for harmful cascade actions was collected by pharmaceutical companies as they developed drugs to decrease those actions. A remaining challenge is to understand the factors that predispose the cascade toward unwanted outcomes and create the need for therapeutic interventions. Such understanding involves recognizing the similar dynamics for dietary n-3 and n-6 nutrients in forming the immediate precursors of the cascade plus the more vigorous actions of the n-6 precursor, arachidonic acid, in forming potent mediators that amplify unwanted cascade outcomes. Tools have been developed to aid deliberate day-to-day quantitative management of the propensity for cascade overactivity in ways that can decrease the need for drug treatments.

## 1. Introduction to Cascade Control

Elucidation of the arachidonic acid cascade [1] began 50 years ago with the simultaneous publication of evidence for enzymatic formation of prostaglandin E2 (PGE2) from the n-6 5,8,11,14-eicosatetraenoic acid, arachidonic acid [2, 3]. The reports were soon accompanied by evidence that two other prostaglandins, PGE1 and PGE3, were formed from the n-6 8,11,14-eicosatrienoic acid and the n-3 5,8,11,14,17-eicosapentaenoic acid, respectively [4]. Another 1964 report described different relative potencies of PGE1 greater than PGE2 greater than PGE3 when controlling the hormone-activated mobilization of fatty acids from adipose tissue [5]. Further research discovered many enzymes in a metabolic cascade that synthesizes a diverse family of bioactive molecules called eicosanoids, including multiple forms of prostaglandins, thromboxanes, and leukotrienes [1]. These potent agents act through selective receptors [6, 7] present on nearly every tissue of the body, giving the cascade an ability to influence nearly every major physiological and pathophysiological event.

Scientific curiosity about molecular mechanisms in the formation and action of the cascade components [1, 8] partnered with the pharmaceutical industry's interest in developing, patenting, and marketing agents that can diminish unwanted pathophysiology. The alliance generated over 175,000 scientific reports cited by PubMed with nearly 5,000 new reports appearing annually for the past 30 years. Understanding the enzymes, cofactors, and receptors that create the health outcomes of the cascade helps identify new targets for drug development [8]. Newly developed agonists and antagonists of the selective receptors [7] continue to attract attention as researchers gain broader understanding of arachidonic acid cascade actions in inflammation and immune function, arthritis, asthma, COPD, cardiovascular diseases (including atherogenesis and thrombosis), metabolic syndrome, back pain, headache, bone density loss, cancer proliferation, Alzheimer's disease, brain development, learning and memory, behavioral disorders, oppositional behaviors, depression, and suicide. This large number of physiological and pathophysiological events indicates the wide scope of conditions in which the balance between benefit and harm from arachidonic acid cascade products constitutes a narrow therapeutic window that this review recognizes as a situation we must manage.

## 2. A Narrow Therapeutic Window for NSAIDs

The first major insight for developing drugs to control the cascade was the recognition that aspirin and nonsteroidal anti-inflammatory drugs (NSAIDs) inhibit the cyclooxygenase-catalyzed conversion of arachidonic acid into prostaglandins [9]. After discovery of the cyclooxygenase product, thromboxane, as a potent derivative that mediates thrombosis [10], aspirin was increasingly used to give benefits by lowering the risk of heart attacks. Small amounts of aspirin gave benefits by irreversibly inhibiting platelet cyclooxygenase. However, adverse side effects and iatrogenic deaths from larger doses of aspirin illustrate the need to recognize how narrow the therapeutic window can be for antithrombogenic agents like aspirin, warfarin, and Plavix. While small doses give benefits by decreasing risk of thrombosis, larger doses cause harm as they impair other needed actions of cyclooxygenase products. Decreasing the risk of unwanted blood platelet activity is now widely achieved by use of “low-dose” aspirin to avoid adverse events that come from blocking beneficial actions of the arachidonate cascade. After a second form of cyclooxygenase was discovered, tens of billions of dollars were spent in developing and marketing various selective “COX-2 inhibitors” with the hope of avoiding the adverse events linked to aspirin use. However, the new agents also had a limited therapeutic window [11], and some were withdrawn from the market.

A challenge for drug developers attempting to control harmful outcomes of the cascade is to find selective agents that will balance the remarkably diverse harmful and beneficial actions of the eicosanoids. The general strategy is to focus on specific events downstream from the initial phospholipase-catalyzed release of eicosanoid precursors and the oxygenase-catalyzed formation of reactive intermediates with the hope of creating more selective inhibition of specific unwanted receptor-mediated outcomes with fewer adverse iatrogenic effects caused by inhibiting beneficial cascade actions. The continuing search for new agonists and antagonists is enhanced by powerful techniques of cloning specific receptors and creating genetic “knock-out” animals [7] to identify explicit downstream effectors that give either beneficial or harmful outcomes.

While the pharmaceutical industry continues searching for drugs to treat and control unwanted actions of the arachidonic acid cascade, an alternate approach is suggested by the evidence that some populations seem less predisposed than others for some cascade-related health disorders [12, 13]. Traditional ethnic food habits may unintentionally provide a lower propensity for harmful cascade outcomes. The 1964 recognition that three different essential fatty acids form three different PGE compounds with different potencies [5] set the stage for considering a strategy of altering the “upstream” precursors of the cascade. Evidence of omega-3 acids competitively inhibiting conversion of n-6 arachidonic acid to prostaglandin [14] showed that the balance of precursor acids in the cascade can control formation rates as well as giving mediators with different receptor responses [5]. Rather than blocking harmful events in the cascade with drugs, people might deliberately create a predisposition for less harmful outcomes and thereby decrease the need for treatments. This review examines the degree to which balancing the n-3 and n-6 20- and 22-carbon highly unsaturated fatty acids (HUFA) that are maintained in the phospholipids of cell membranes can control beneficial and harmful outcomes of the arachidonic acid cascade.

## 3. Precursor-Based Propensities and Predispositions

**Figure 1:**
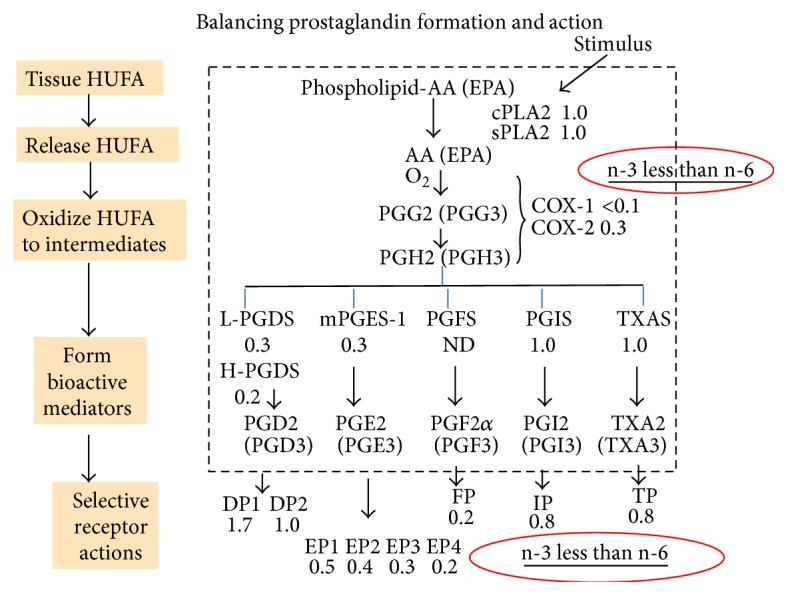
Balancing prostaglandin formation and action. The prostaglandin part of the arachidonic acid cascade begins with a stimulated phospholipase A2 releasing precursor HUFA from membrane phospholipids. The relative intensity of reaction for n-3 and n-6 mediators is shown as a ratio next to the interacting enzyme or receptor.

The relative intensity of action by the n-3 eicosapentaenoic acid (20:5n-3) relative to that for the n-6 eicosatetraenoic acid (20:4n-6) in the prostaglandin part of the cascade is shown in Figure 1 as a ratio next to the enzymatic step or specific receptor. The initial step of phospholipase release of the precursor does not discriminate between the n-3 and n-6 structures, giving ratios of 1.0 for both cytosolic (cPLA2) and secretory (sPLA2) hydrolase actions. In contrast, both cyclooxygenases, COX-1 and COX-2, react more slowly with the n-3 than the n-6 substrate, giving cascade-mediated actions that are less intense with the n-3 precursor [15]. Different proportions of n-3 and n-6 precursors in cellular membrane lipids give different intensities of n-6 agonist action, and weak n-3 agonist action inevitably becomes an antagonist of the more potent n-6 agonist.

This difference in actions occurs also with the synthases that form PGD and PGE, ensuring that supplies of those prostaglandins at their specific cell-membrane receptors will tend to be less intense for n-3 than n-6 eicosanoids. Finally, the selectively lower responses by EP and FP receptors with n-3 mediators moderate even further any predisposition or propensity for excessive actions of the arachidonic acid cascade. The consequence of different cascade responses to n-3 and n-6 acids was summarized in a 2014 review in terms of a therapeutic window for dietary essential n-6 nutrients that is very narrow, and its margin of safety is widened by eating n-3 nutrients [16].

**Figure 2:**
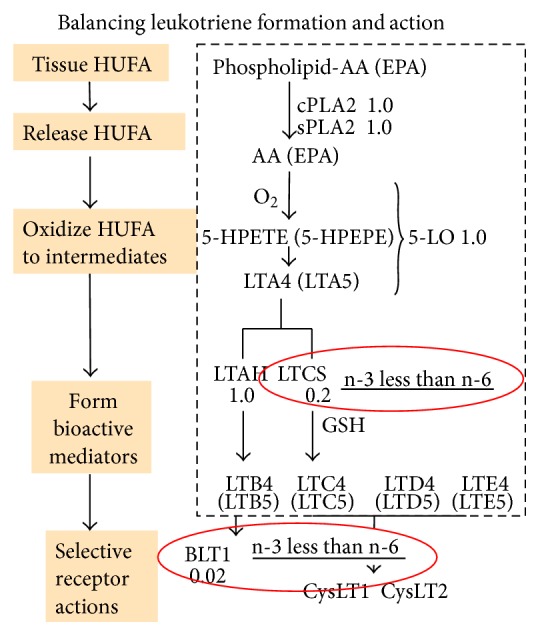
Balancing leukotriene formation and action. The leukotriene part of the arachidonic acid cascade begins with a stimulated phospholipase A2 releasing precursor HUFA from membrane phospholipids. The relative intensity of reaction for n-3 and n-6 mediators is shown as a ratio next to the enzyme or receptor.

For the leukotriene portion of the cascade shown in Figure 2, the first two steps, PLA2 and 5-lipoxygenase (5-LO), are relatively indiscriminate, whereas the leukotriene C synthase (LTCS) forms cysteinyl leukotrienes 10-fold [17] to 20-fold [18] less vigorously with n-3 than n-6 LTA intermediates. The lower intensity of LTC formation allows a higher proportion of n-3 in HUFA to lower the intensity of chronic bronchopulmonary events seen in asthma and chronic obstructive pulmonary disease (COPD). Additionally, the LTB receptor (BLT-1) that mediates important immune-inflammatory events responds 50-fold less vigorously to n-3 LTB5 than the potent n-6 LTB4 [19], making the development of severe inflammatory loci less intense when the HUFA balance has more n-3 HUFA.

The examples provided above illustrate how both n-6 and n-3 precursors give active mediators in the arachidonic acid cascade. However, the more intense responses from n-6 mediator actions increase the propensity for cascade overreactions that shift conditions from normal physiology to pathophysiology when the n-6 precursor is the dominant HUFA available. As a result, arranging for n-3 HUFA to be a greater proportion of the HUFA can moderate cascade-mediated events and widen the therapeutic window for dietary n-6 precursors. Of course, the cascade is not the only way that HUFA-based events affect human health. Endocannabinoids, arachidonyl glycerol and arachidonoyl ethanolamide, are HUFA-based bioactive lipids that act through selective receptors, CB1 and CB2. While some evidence suggests that arachidonoyl glycerol may be the biologically relevant mediator [20], results with fatty acid amide hydrolase inhibitors [21] indicate that both n-3 and n-6 HUFA ethanolamides may have important beneficial actions.

Although some studies show that CB1 and CB2 receptors have high structural specificity for the n-6 structure with a double bond at position 5 [20, 22, 23], the degree to which competing n-3 and n-6 forms of the ester and amide types of mediators influence health outcomes remains uncertain. Similarly, n-6 lipoxins [24] formed from arachidonic acid by multiple lipoxygenase actions bind the ALX/F receptor [25] and counteract some unwanted events mediated by the cascade. Further research studies will assign the degree to which various endocannabinoid and lipoxin actions contribute to major human health conditions and whether the balance of n-3 and n-6 HUFA influences their actions. Another group of n-3 HUFA-based bioactive mediators includes resolvins, protectins, and maresins [26] which counteract and help resolve inflammatory events promoted by the n-6 cascade mediators. With all of the mediator actions noted above, the same strategy of arranging for n-3 HUFA to be a greater proportion of tissue HUFA will moderate unwanted cascade-mediated health conditions caused by overabundant n-6 precursor availability and action. The degree of dominance of n-6 HUFA among the HUFA available for tissue responses can be called “HUFA balance,” and it provides a useful biomarker that can be expressed as either the %n-6 in HUFA or the %n-3 in HUFA. Because the n-3 and n-6 acids are dietary essentials, accumulating a desirable HUFA balance in tissues inevitably depends on the average proportions of essential n-3 and n-6 nutrients provided by personal food choices. Examples of the impact of ethnic food habits on HUFA balance and health outcomes are noted below.

## 4. Food Habits Affect the Intensity of Cascade Outcomes

The 1979 report [12] relating food choices to the balance in actions of prostacyclin (PGI) and thromboxane (TXA) described a striking difference in the relative amounts of n-3 to n-6 precursors in blood phospholipids of Danes (0.2 n-3 to 8.0 n-6) and Eskimos (7.1 n-3 to 0.8 n-6). Subsequent increasingly detailed analyses have described a HUFA balance of 32% n-6 in HUFA in Greenland [27] and 44% [28] or 41% to 38% in Canadians at Nunavik [29]. A more recent survey at Nunavik showed different age groups with blood HUFA balance of 52% n-6 in HUFA for 45–74 years of age, 61% for 35–44, 68% for 25–34, and 72% for 18–24 [30]. The biomarker differences reflect the rapidly changed lifestyles among Arctic people as they acquire access to commercially prepared foods not typical for their traditional lifestyle [12]. The long-recognized low incidence of heart attacks among these people is not attributable to low blood cholesterol [31]. Rather, it likely is due to high tissue proportions of the moderating omega-3 precursors of the arachidonic acid cascade that blunt the predictive role of the food energy biomarker, blood cholesterol (see Figure  1 in [16]).

Japanese people also experienced a change in lifestyle in the past 50 years as “Western-style” processed foods displaced traditional foods and shifted the balance of arachidonate cascade precursors in their tissues [13]. The proportion of n-6 in HUFA has been progressively higher for younger Japanese generations until values for the youth approach those of American youth. The %n-6 in HUFA was near 35% for Japanese born in 1938, 42% for 1950, and 60% for 1974 [32]. The long-recognized low death rates from prostate cancer among Japanese octogenarians [33] rose steadily from 50 to 200 (per 100,000 people) between 1960 and 2000 while prostate cancer death rates for Americans (with a consistently higher %n-6 in HUFA near 78%) remained relatively unchanged at 500.

During this time, corresponding Italian prostate cancer mortality rates rose from 200 to 400 [33] while changes from traditional Mediterranean food habits were observed [34, 35]. In 2013, biomarker values near 84% were reported for 2- to 9-year-old Italian children, whereas the average was 78% for 40–59-year-old adults [36] and 79-80% for Italians in a multicenter study [37]. Residents of northern Italy had values of 80% n-6 in HUFA, whereas those on the islands of Sardinia and Sicily had values near 73% [38]. The biomarker was 67% n-6 in HUFA for residents of Barcelona on the Mediterranean coast [39], and it was 72% when inland Spanish communities were included [40]. Definitions of the “traditional Mediterranean Diet” become increasingly imprecise when children in Portugal have 78% n-6 in HUFA compared to 76% in Germany [35].

The biomarker of %n-6 in HUFA indicates the propensity for arachidonate cascade overreactions and is a useful indicator for health risk assessment. For example, the incidence of heart attacks for the quintile of Americans maintaining about 62% n-6 in HUFA was nearly one-half of that for those with median values near 80% [41]. Cross-cultural comparisons showed a very close association of CHD mortality with values from 32% to 80% n-6 in HUFA [41]. Knowing about n-6 mediators of inflammatory atherogenesis and of platelet-mediated thrombosis makes a change of 10% to 20% in this biomarker an important aspect in health conditions. Blasbalg et al. described the dramatic rise in n-6 linoleic acid consumption in the USA during the late 20th century [42]. The change reflects a widespread insertion of vegetable oils into many “modern” processed foods. That change was assessed by one research group [43] in these terms: “The widespread consumption of diets with more than 2% energy as LA should be recognized for what it is—a massive uncontrolled human experiment without adequate rationales or proven mechanisms.” A constructive alternative to this situation would be to apply quantitative knowledge of the metabolic dynamics of dietary n-3 and n-6 nutrients to moderate the current propensity for unwanted overactive arachidonic acid cascade events.

## 5. Metabolic Dynamics in Balancing Tissue HUFA Proportions 

The 18-carbon n-3 linolenic and n-6 linoleic acids in foods are major precursors of the n-3 and n-6 HUFA that accumulate in tissue phospholipids and eventually form eicosanoids. The n-3 and n-6 nutrients compete for the same enzymes with similar hyperbolic dynamics during the desaturation and elongation steps that form the HUFA [44–46]. Steep dose-response curves for n-3 and n-6 18-carbon essential nutrients providing tissue 20- and 22-carbon HUFA (see Figure  4 in [47]) describe the response when those nutrients are supplied in the range of 0 to 1 percent of daily food energy (en%). When supplied in amounts above 1 or 2 en%, a blunted dose-response relationship has the hyperbolic “plateau” characteristic of all saturable enzyme-catalyzed metabolic processes. The lack of a continuing linear response does not indicate that the precursor does not form the product. Rather, it is a reminder that dose-response dynamics need to be interpreted in terms of the range of substrate supply that is provided. Interpretations of results from diet-tissue studies can be improved by acknowledging that the sensitive dose-response range for accumulating n-6 HUFA from linoleic acid is below 0.5%en [16].

Another caution arises when n-3 linolenate is ingested in mixtures with severalfold greater amounts of n-6 linoleate. In this case, the indiscriminate enzymes inevitably react in proportion to the substrate supplied and make the n-3 nutrient appear less productive than the competing n-6 nutrient in forming and accumulating tissue HUFA [45]. This effect was evident in a large meta-analysis [48] that was limited to only additions of an n-3 fatty acid to diets that had much more n-6 than n-3 nutrient in all cases evaluated. The limited data led to limited interpretations. However, the seldom described or discussed converse condition of eating more n-3 than n-6 precursor clearly gives more accumulated n-3 than n-6 HUFA [45, 49]. The n-3 linolenic acid is not a poor substrate for forming HUFA. A 2014 review of the dynamics of essential fatty acid actions and tissue HUFA formation [16] noted that dietary intakes of linoleic acid between 0.3 and 0.5 percent of food energy (en%) prevent signs of essential fatty acid deficiency and maintain tissue arachidonate above 50% of HUFA [44, 45, 49]. Importantly, lower values for the %n-6 in HUFA are associated with less inflammatory conditions [50] and cardiovascular events [41]. Lower values for HUFA balance are made possible by competing dietary n-3 nutrients that keep the tissue HUFA balance below 50% n-6 in HUFA. In the absence of n-3 nutrients, very small amounts (0.5 percent of daily calories) of the essential n-6 nutrient, 18:2n-6, can give a HUFA balance above 50% n-6 in HUFA, approaching values that are clearly associated with greater harm. Thus, the n-6 nutrient has a very narrow to almost nonexistent therapeutic window. Fortunately, the window of safety is widened by competing n-3 nutrients [16].

Some foods contain already formed n-3 and n-6 HUFA that compete with newly formed HUFA for storage in cellular phospholipids. The nonlinear competing interactions were described in 1992 by an empirical hyperbolic equation that fit data for rats, mice, and humans [51]. The general equation estimated well the observed balance of tissue n-3 and n-6 HUFA that was maintained by known daily intakes of n-3 and n-6 nutrients for nearly 4,000 people in 92 subject groups in 34 different studies from 11 different countries [52]. The similarity of the relatively indiscriminate dynamics for lipid metabolism among mammals reflects a well-recognized biological phenomenon of similar enzymes coded by similar housekeeping genes that did not vary much in structure or function during the evolution of diverse species.

Nevertheless, genetic adaptation has occurred for a human-specific haplotype defined by 28 closely linked single-nucleotide polymorphisms (SNPs) which affects the biosynthesis of HUFA from the 18-carbon precursors [53]. Haplotype D gives faster fatty acid desaturase activities (FADS1 and FADS2), making the newly synthesized HUFA more closely reflect the proportions of the 18-carbon precursors (and reflect less the dietary HUFA proportions). Interestingly, the “fast” haplotype D shows evidence of positive selection in African people and is less frequent outside of Africa [53, 54]. As a result, many African Americans living in a current food environment with high intakes of n-6 linoleic acid may now accumulate a higher %n-6 in HUFA [55, 56] than occurring in the African environment that favored selection of the alleles that form the D haplotype. With a genetic propensity to convert the USA high proportion of n-6 to n-3 PUFA into higher proportions of arachidonate in tissue HUFA, African Americans illustrate more clearly than Caucasians the need to lower intakes of n-6 PUFA and decrease the HUFA balance of %n-6 in HUFA to avoid excessive actions of the arachidonate cascade.

## 6. Choosing Foods That Lower the Propensity for Cascade Overreaction

The balance of tissue HUFA maintained by daily food habits is readily measured by simple gas chromatographic analysis of a finger-tip blood-spot [57, 58]. As noted above, values for the HUFA balance range from 25% to 85% n-6 in HUFA depending on a person's average daily food habits. This health risk assessment biomarker relates predictably to the average daily balance of n-3 and n-6 nutrients (expressed as en%) in the foods that are routinely eaten [16, 51]. Several tools were developed to show people how the n-3 and n-6 nutrient supply relates to the tissue HUFA precursors of the arachidonate cascade. A simple spreadsheet calculator [59] embeds the empirical competitive hyperbolic relationship [60] to help clinicians design dietary interventions that can create sufficient differences in HUFA balance between intervention and control groups.

The equation [60] is also combined with specific nutrient data from the USDA Nutrient Database [61] to give an interactive computer program, KIM-2 [62], to give estimates of the likely HUFA balance that would result from continued ingestion of foods in the plan. Tissue lipid pools need weeks and months to equilibrate, and nutritionists advise having many different daily plans during that time to maintain palatability. Examples of daily plans that give HUFA balances of 15%, 26%, 35%, 50%, 63%, 71%, and 91% n-6 in HUFA are described (chapter 19 in [63]).

To see more quickly the likely impact that a food item will have on the eventual HUFA balance, eleven different n-3 and n-6 nutrients in a food item (expressed as mg/Cal) are combined into a single Omega 3-6 Balance Score for each food item [64]. Foods with positive scores increase the proportion of n-3 in HUFA, whereas foods with negative scores increase the proportion of n-6 in HUFA. The scores range in values from +100 to −200, and many common vegetables, fruits, and dairy products have values near 0. The calorie-weighted average score for a day's food relates linearly to the health risk assessment biomarker predicted by the software [62]. As average daily food scores range from +3 to −7, the corresponding blood biomarker value ranges from 25% to 85% n-6 in HUFA [64]. Deleting foods with high negative scores and adding foods with positive scores lower the %n-6 in HUFA. Significant positive health benefits came in a randomized controlled clinical trial that used this approach for three months and lowered the biomarker value from 77% to 61% n-6 in HUFA [65]. Such results parallel the finding that CHD deaths were nearly one-half for the quintile of Americans maintaining about 62% n-6 in HUFA compared to those with median values near 80% [41].

Lowering the propensity for arachidonate cascade overreaction is important for most large USA employers that have large financial losses due to employees' health-related absenteeism, presenteeism, and medical and pharmacy expenses [66]. Many health conditions that cause major financial losses [67] are made worse by n-6 mediators in arachidonate cascade overreactions (Table 1). A simple wellness plan that informs employees of their health risk status from finger-tip blood-spot assays and informs them of Omega 3-6 Balance Scores of common foods may help employees voluntarily shift their HUFA balance toward the lower values of %n-6 in HUFA which have a lower propensity for arachidonate cascade overreactions [16]. An Omega 3-6 Balance Score App [68] lists over 5,000 foods in a searchable format.

The ease with which a voluntary shift can be made is illustrated with the 100 foods most frequently eaten by Americans [69] which have an average Omega 3-6 Balance Score near −6 [16, 64]. Removing the ten foods with the most negative scores gave 90 items with an average value near −3 (a value associated with traditional Mediterranean meals). The removed foods were not typically eaten 100 years ago in Mediterranean households, and their recent use is likely a reason for current measures of HUFA balance for Mediterranean people to be near 75% to 80% rather than 63%. Finding food combinations that shift the average daily Omega 3-6 Balance Score a few points more positive may decrease the HUFA balance to a 10% to 20% lower value and lower the unwanted propensity for arachidonate cascade overreactions.

## 7. Conclusion

Omega-3 nutrients play an important role in moderating the inherent propensity for arachidonic acid cascade overreactions when n-6 mediators dominate. The unintended consequences of eating foods that create such conditions can be prevented by combining knowledge about the explicit balance of n-3 and n-6 nutrients in each food item with knowledge of the dynamics of fatty acid metabolism and the different intensities of n-3 and n-6 eicosanoid actions. Tools are freely available for making informed food choices that can prevent serious health conditions caused by cascade overreactions.

## Figures and Tables

**Table 1:** Prevalence of health conditions causing major USA health care costs. The 25 most prevalent conditions in a large occupational medicine study [67] are shown with the ten having the most annual cost ranked in order. The overall annual costs include expenses from medical, pharmacy, absenteeism, and presenteeism aspects.

Health condition prevalence		Cost
	Rank	Rank
Depression	6	1
Obesity	2	2
Arthritis	4	3
Back and neck pain	9	4
Anxiety	7	5
GERD	5	6
Allergy	1	7
Other cancers	19	8
Other chronic pains	17	9
Hypertension	3	10

Asthma	8	
Migraine	10	
Sleeping problem	11	
Irritable bowel	12	
Fatigue	13	
Headache	14	
Diabetes	15	
Bladder/urinary	16	
Ulcer	18	
Coronary heart disease	20	
Osteoarthritis	21	
Skin cancer	22	
Bronchitis/emphysema	23	
Congestive heart failure	24	
COPD	25	
